# Unraveling Polycyclic Aromatic Hydrocarbon-Triggered Reactive Oxygen Species’ Generation in Maize Rhizosphere: Coupled Biotic–Abiotic Mechanism

**DOI:** 10.3390/life16071136

**Published:** 2026-07-08

**Authors:** Xiaoling Xu, Chuanxiang Li, Jinbo Liu, Jian He, Yongxiu Sun, Jian Wang

**Affiliations:** 1 School of Petroleum Engineering and Environmental Engineering, Yan’an University, Yan’an 716000, China; xuxiaoling0309@163.com (X.X.); 15634129251@163.com (C.L.); hej19@lzu.edu.cn (J.H.); wangjian@yau.edu.cn (J.W.); 2 Yan’an Key Laboratory of Ecological Restoration and Carbon Sequestration Regulation, Yan’an 716000, China; yongxiusun@yau.edu.cn

**Keywords:** polycyclic aromatic hydrocarbons, rhizosphere, maize, reactive oxygen species, root exudates

## Abstract

Reactive oxygen species (ROS) are critical drivers of redox-associated biogeochemical processes within the rhizosphere, yet the mechanisms of their generation under contaminant stress remain poorly understood. A 24-day pot cultivation experiment with four treatments (control, naphthalene, phenanthrene, and anthracene) was conducted to investigate how polycyclic aromatic hydrocarbons (PAHs) alter the production of three kinds of ROS (e.g., O_2_^•−^, H_2_O_2_, and ^•^OH) in the maize rhizosphere. PAHs promoted the production of rhizosphere ROS, and the promotion effects were compound-dependent, following the order of anthracene > phenanthrene ~ naphthalene. The increases in O_2_^•−^ content were 55.6%, 14.3%, and 17.9% under anthracene, phenanthrene, and naphthalene treatments. The H_2_O_2_ content was enhanced by 58.6% under anthracene treatment, 10.4% under phenanthrene treatment, and 15.4% under naphthalene treatment. The ^•^OH concentrations increased by 62.5%, 21.1%, and 0.5% under anthracene, phenanthrene, and naphthalene exposure, respectively. Importantly, the variations in rhizosphere ROS’ content simultaneously fluctuated with stem length, photosynthetic rates, root exudates, dissolved organic carbon (DOC), water-soluble phenols, and enzymes activities induced by PAHs stress. Statistical analysis suggested PAH stress enhanced maize biomass (particularly stem growth), thereby improving photosynthetic efficiency and thus stimulating root exudate release. Root exudates could promote water-soluble phenol and DOC release and enhance microorganism reproduction, thereby mediating abiotic ROS’ production via electron transfer and biotic ROS’ production via extracellular release. These findings clarify the response of rhizosphere ROS to PAHs stress, providing valuable insights for rhizosphere-ROS-mediated remediation of soil pollutants.

## 1. Introduction

Reactive oxygen species (ROS) occur in varied environmental matrices [1,2,3,4,5,6], where they drive carbon dynamics, facilitate nutrient cycling, mediate contaminant degradation, and modulate the physiological functions of organisms [7,8,9,10,11]. Current studies have revealed that the soil rhizosphere is an emerging hotspot for ROS production. Using microfluidic chip setup, Dai et al. first demonstrated ROS in situ production in rice rhizosphere, which displayed diel fluctuation [12]. Recent work uncovered root exudates, active microbial metabolism, and mineral-driven redox reactions jointly sustain continuous ROS production in rhizosphere [9,13]. Accordingly, it is plausible to infer that ROS are widely prevalent in the rhizospheres of different plants, and revealing that the sources, spatial patterns, and environmental roles of ROS within the rhizosphere are essential for advancing scientific progress in soil biogeochemistry.

The rhizosphere represents a dynamic interface characterized by intense biogeochemical activities driven by roots [14,15]. Compared to bulk soil, the rhizosphere is characterized by unique pH values, superior amounts of organic substances, enzyme activity, and microbial abundance [16,17]. Notably, these attributes are highly sensitive to environmental factors. Studies have verified that root type, soil moisture, and temperature modulate rhizosphere ROS’ production, yet how contaminants reshape rhizosphere ROS’ dynamics remains poorly resolved. Pollutants can inhibit plant growth and development, reducing root physiological activities such as water content and respiration [18,19,20]. Additionally, the interaction between plants and pollutants regulates root exudates’ release and composition, which in turn affect enzyme activities and recruitment and enrichment of specific microbial communities [21]. Given that such alterations in plant physiology and root exudation patterns are key determinants of physicochemical and biological characteristics [9,13,22], they are likely to play a crucial part in regulating rhizosphere ROS’ generation. Accordingly, we hypothesized that pollutant stress may influence ROS’ formation in the rhizosphere, the occurrence of which could have significant implications for rhizosphere biogeochemical processes. More importantly, rhizosphere ROS can drive the degradation of persistent organic pollutants [12,13], making them key players in phytoremediation. Harnessing rhizosphere ROS for in situ contaminant degradation represents a green, cost-effective, and sustainable remediation strategy. Yet, how pollutants control ROS production remains a missing link for developing green remediation strategies.

Polycyclic aromatic hydrocarbons (PAHs) are ubiquitous persistent organic pollutants in soils, primarily derived from anthropogenic-related incomplete combustion and petroleum-related activities [23,24]. Major entry pathways include atmospheric dry/wet deposition from vehicle emissions, coal/biomass burning and industrial coking, irrigation with contaminated water, and application of polluted organic amendments including manure and sludge [25,26]. As highly hydrophobic compounds, PAHs exhibit strong binding affinity with soil organic matter and clay minerals. Although PAH stress is known to affect plant physiology and rhizosphere microbial communities [25], critical knowledge gaps remain. The molecular-structure-dependent mechanisms by which different PAHs orchestrate ROS’ production via integrating plant physiological and rhizosphere microbial processes remain critically underexplored. For example, how do PAHs with varying ring structures (e.g., two-ring naphthalene (NAP), three-ring phenanthrene (PHE) and anthracene (ANT)) differentially affect maize physiology, root exudation chemistry, and microbial activity to collectively drive ROS’ generation? We hypothesized that ANT, due to its higher hydrophobicity, stronger root surface adsorption and greater phytotoxicity, would induce larger production of dissolved organic carbon, water-soluble phenols and microbial extracellular enzymes, leading to significantly higher rhizosphere ROS content than PHE and NAP.

To test these hypotheses, maize, an extensively utilized model plant, was applied to determine potential production of ROS in its rhizosphere under the stress of PAHs. The principal purposes were (i) to explore the changes in rhizosphere ROS including O_2_^•−^, H_2_O_2_, and ^•^OH under the stress of different PAH molecules (e.g., NAP, PHE, and ANT), (ii) to offers key insights into the effects of PAHs on ROS’ formation by examining maize biomass, photosynthetic rates, root exudates, soil redox-active components, and enzyme activities, and (iii) to unravel the mechanisms underlying ROS’ generation under PAH stress using structural equation modeling (SEM). These findings shed new light on the effects of PAHs on ROS generation in rhizosphere and offer significant implications for advancing phytoremediation strategies associated with ROS.

## 2. Materials and Methods

### 2.1. Reagents and Soils

For the reagents employed in this research, refer to Text S1 (Supplementary Material). The soil samples used were consistent with those described in our prior work [9]. Detailed information on their pretreatment, analytical methods, and physicochemical characteristics is available in our previous work.

### 2.2. Preparation of PAH-Contaminated Soils

A laboratory soil-spiking method was employed to simulate environmentally relevant PAH contamination. Specifically, we added 50 g of clean soil to a 250 mL Erlenmeyer flask and then incorporated 25 mL of acetone solution spiked with 200 mg L^−1^ of PAHs. Thereafter, we shook the mixture at 70 rpm for 2 d on a rotary shaker to ensure complete acetone evaporation and uniform contaminant dispersion. Finally, we introduced 450 g of clean soil to the flask and thoroughly mixed it for uniform PAH distribution, corresponding to an initial concentration of 10 mg kg^−1^. This concentration was selected based on typical environmental levels of PAHs in contaminated agricultural soils [24,25,26]. To verify spiking efficiency, triplicate subsamples of each PAH-contaminated soil were extracted by Soxhlet using a dichloromethane/n-hexane mixture (1:1, *v*/*v*), purified on silica gel columns, and analyzed by HPLC. The results showed that the measured concentrations of NAP, PHE, and ANT were all within 95–105% of the nominal 10 mg kg^−1^, indicating spiking accuracy.

### 2.3. Variations in ROS Under PAH Stress

ROS content under PAHs stress was investigated to clarify PAHs’ effect upon ROS’ production by using pot experiment. A total of 500 g of PAHs-contaminated soils was put into plastic pots (length × width × depth, 30 × 20 × 15 cm). Thereafter, 2 uniform maize (*Zea mays cv. Zhengdan 958*) seeds were inoculated into each pot following sterilization. Specifically, maize seeds were surface-sterilized by immersion in 3% (*v*/*v*) H_2_O_2_ for 10 min with gentle agitation, followed by thorough rinsing with sterile deionized water to remove residual H_2_O_2_. Sterilization efficacy was verified by the absence of colony growth on LB agar plates inoculated with 100 μL of the final rinse water after incubation at 28 °C for 48 h. Following germination, we trimmed seedlings to one plant and pre-cultured them for 1 week to promote root development. Maize was then grown in a climate chamber with a photosynthetic photon flux density of 1000 μmol m^−2^ s^−1^, a temperature of 20 ± 1 °C, and a photoperiod of 14/10 h (light/dark). Soil moisture was maintained at approximately 60% of field capacity utilizing periodic addition of distilled water. As contents of ROS like O_2_^•−^ and H_2_O_2_ in rhizosphere of maize attained their maximum and steady levels at 24 d [9], the batch incubation experiment (9 replicates × 4 treatments = 36 pots total) was halted after 24 d of incubation. The roots were gently shaken to remove loosely adhered bulk soil. The soil tightly attached to the root surface (approximately 0–2 mm from the root) was carefully collected by brushing and defined as rhizosphere soil. All 36 experimental pots were randomly rearranged and repositioned every three days inside the climate chamber to eliminate uneven light, temperature and humidity bias across the cultivation space. Soil sampling order for each replicate pot was also fully randomized during harvest to avoid operational bias. The soil samples collected were rapidly examined for ROS content and other physicochemical attributes.

### 2.4. Maize Biomass, Root Exudates and Photosynthetic Rates

After rhizosphere soil collection, maize root and stem lengths were measured, and root exudates were quantified; the specific procedures are given in Text S2. Given that PAH stress enhances maize biomass and root exudate release, we measured photosynthetic rates to investigate potential underlying connections. Briefly, net photosynthetic rate was measured in middle leaves using LI-6400 gas exchange system after 24 d of maize cultivation.

### 2.5. ROS Measurement

The contents of ROS were quantitatively measured by various chemical probes. Specifically, the O_2_^•−^ content was examined by using 2,3-bis(2-methoxy-4-nitro-5-sulfophenyl)-2Htetrazolium-5-carboxanilide (XTT), which reacts with O_2_^•−^ to produce XTT formazan [27]. H_2_O_2_ content was detected by using hydrogen peroxide assay kit. The determination of ^•^OH content was conducted using benzoic acid, which reacts with ^•^OH to form 4-hydroxybenzoic acid [28]. Specific protocols for the measurement of O_2_^•−^, H_2_O_2_, and ^•^OH are described in Text S3. It should be noted that the ROS’ concentrations reported herein were quantified using indirect chemical probes, which, while widely employed in soil studies, have inherent limitations. First, these probes may react with other oxidizing species present in the rhizosphere, potentially leading to overestimation. Second, the probes measure cumulative ROS’ production over the extraction period rather than instantaneous concentrations, and the extraction procedure may itself induce ROS generation.

### 2.6. Measurement of Rhizosphere Soil Physicochemical Properties and Enzymes Activity

We quantified Fe(II), dissolved organic carbon (DOC), and water-soluble phenols’ concentrations in rhizosphere soil; for specific methods, refer to Texts S4–S6. Furthermore, the activities of microbial-activity-associated enzymes such as urease and dehydrogenase and ROS-linked enzyme (e.g., catalase) were assessed (Text S7).

### 2.7. Statistical Analysis

All experiment results are provided as mean ± SD. Prior to analysis, data were tested for normality using the Shapiro–Wilk test and for homogeneity of variances using Levene’s test. When data met these assumptions, the differences among treatments were assessed by ANOVA, followed by LSD test, and significance was judged by *p* < 0.05. To identify the pivotal factors influencing ROS production, correlation analysis was conducted with Origin 2025. SEM was conducted with Amos 20.0 software (IBM Corporation Software Group, Somers, NY, USA) to clarify potential ROS’ production mechanisms. The model incorporated PAH stress as the exogenous variable, maize photosynthetic rate, root exudates, dissolved organic carbon (DOC), water-soluble phenols, and microbial activity as intermediate endogenous variables- and three ROS species (O_2_^•−^, H_2_O_2_, and ^•^OH) as final response variables. Prior to modeling, all data were log-transformed to meet the normality assumption. Model fitness was evaluated via multiple standard indices, including chi-square (χ^2^), degrees of freedom (df), *p*-value, root mean square error of approximation (RMSEA), and Akaike information criterion (AIC). Models with *p* > 0.05, RMSEA = 0, and low AIC values were regarded as well-fitted. Standardized path coefficients were calculated to compare the relative strength of each causal pathway. The specific model output parameters are provided in Text S8.

## 3. Results

### 3.1. Effects of PAH Exposure on Generation of Rhizosphere ROS

**Figure 1:**
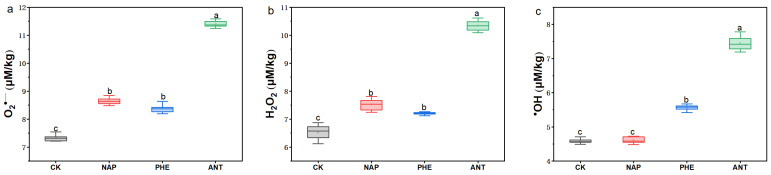
Variation in (**a**) superoxide anion (O_2_^•−^), (**b**) hydrogen peroxide (H_2_O_2_), and (**c**) hydroxyl radical (^•^OH) in rhizosphere soil following PAH addition. Control contained no PAHs, whereas ANT, PHE and NAP treatments were amended with 10 mg kg^−1^ of anthracene, phenanthrene, and naphthalene, respectively. Error bars represent standard error of mean (*n* = 9). Significant differences among treatments were analyzed via one-way ANOVA followed by LSD test. Distinct lowercase letters above bars denote significant differences at *p* < 0.05.

As exhibited in Figure S1, the fluorescence distribution pattern is consistent with the shape of the maize root system, indicating the production of ROS in the maize rhizosphere. Moreover, all tested PAHs greatly propelled three ROS’ generation in rhizosphere soils (Figure 1, *p* < 0.05), with ANT exerting the most prominent effect. Specifically, in the control group without PAH addition, the concentrations of O_2_^•−^, H_2_O_2_, and ^•^OH in rhizosphere soils were 7.33, 6.52, and 4.59 μM/kg, respectively. The concentrations of O_2_^•−^, H_2_O_2_, and ^•^OH increased to 8.64, 7.53, and 4.61 μM/kg, 8.38, 7.20, and 5.56 μM/kg, and 11.40, 10.34, and 7.46 μM/kg, respectively, with adding ANT, PHE and NAP. Correspondingly, relative increments compared to the control group were 17.9%, 15.4%, and 0.5% for NAP, 14.3%, 10.4%, and 21.1% for PHE, and 55.6%, 58.6%, and 62.5% for ANT. Statistical analysis further confirmed that rhizosphere ROS concentration in ANT-amended soil was significantly higher than that in NAP and PHE soils (*p* < 0.05), highlighting distinct differences in ROS-inducing capacities among these PAHs. Overall, three PAHs elevated rhizosphere ROS’ contents with distinct compound-specific effects, following the order of ANT > PHE ~ NAP.

### 3.2. Effects of PAH Exposure on Biomass, Photosynthetic Rates and Root Exudates

**Figure 2:**
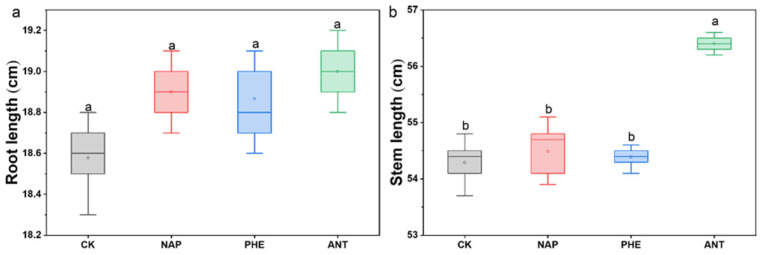
Effects of PAH addition on (**a**) root length and (**b**) stem length of maize. Error bars represent standard error of mean (*n* = 9). Control contained no PAHs, whereas ANT, PHE and NAP treatments were amended with 10 mg kg^−1^ of anthracene, phenanthrene, and naphthalene, respectively. Significant differences among treatments were analyzed via one-way ANOVA followed by LSD test. Distinct lowercase letters above bars denote significant differences at *p* < 0.05.

The physicochemical and biological characteristics in the rhizosphere generally correlated with the differences in maize biomass. Accordingly, the biomass of maize under PAH stress was measured to explore the potential link between PAH-induced ROS’ changes and plant growth responses. Referring to Figure 2a, root length exhibited a slight but statistically insignificant increase under PAH addition (*p* > 0.05), ranging from 18.6 to 19.0 cm. Similarly, although PAH addition exhibited a slight promoting effect on stem length, the enhancement was not pronounced (Figure 2b, *p* > 0.05), except in the case of the ANT treatment (Figure 2b, *p* < 0.05). Specifically, stem length without PAH addition was 54.3 cm. When adding ANT, PHE, and NAP, stem length increased to 54.5, 54.4 and 56.4 cm, respectively. Overall, the addition of PAHs enhanced maize biomass, which may induce ROS’ generation in the maize rhizosphere.

**Figure 3:**
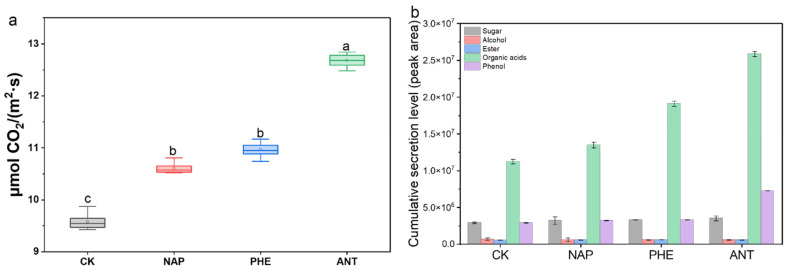
PAH addition impact on (**a**) photosynthetic activity and (**b**) cumulative secretion of compounds in maize. Control contained no PAHs, whereas ANT, PHE and NAP treatments were amended with 10 mg kg^−1^ of anthracene, phenanthrene, and naphthalene, respectively. Error bars represent standard error of mean (*n* = 9). Significant differences among treatments were analyzed via one-way ANOVA, followed by LSD test. Distinct lowercase letters above bars denote significant differences at *p* < 0.05.

To further explore this mechanism, changes in photosynthetic rate and root exudates under PAH exposure were also investigated. As shown in Figure 3a, the net photosynthetic rates under PAH addition exhibited an upward trend compared with the control. Notably, the ANT treatment showed the highest net photosynthetic rate, representing a significant 1.34-fold increase over the control. This enhancement may have been related to PAH stress, which could regulate photosynthesis-related genes’ expression in maize. Similarly, PAH addition dramatically rose total root exudate content (*p* < 0.05), with the most pronounced effect observed under ANT exposure (Figure 3b). Although the overall chemical composition of exudates remained largely consistent across treatments (Tables S1 and S2), distinct differences emerged in the abundance of specific compounds. Specifically, the abundance of organic acids increased markedly under ANT exposure (approximately 2.3-fold compared to the control), with ~1.7- fold and ~1.2-fold increases under PHE and NAP addition. Likewise, phenolics showed the greatest elevation under ANT stress, up to 2.5-fold.

### 3.3. Effects of PAH Exposure on Redox Components and Enzymatic Activity in Rhizosphere

**Figure 4:**
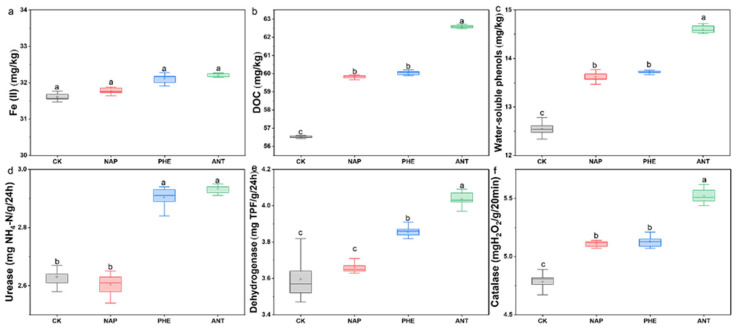
PAH addition’s impact on (**a**) Fe(II), (**b**) dissolved organic carbon (DOC), (**c**) and water-soluble phenol content and (**d**) urease, (**e**) dehydrogenase, and (**f**) catalase activity. Control contained no PAHs, whereas ANT, PHE and NAP treatments were amended with 10 mg kg^−1^ of anthracene, phenanthrene, and naphthalene, respectively. Error bars represent standard error of mean (*n* = 9). Significant differences among treatments were analyzed via one-way ANOVA, followed by LSD test. Distinct lowercase letters above bars denote significant differences at *p* < 0.05.

As exhibited in Figure 4a, the Fe(II) content remained largely unchanged with the addition of PAHs, keeping at a constant level of 31.60~32.19 mg/kg. Comparatively, PAHs induced an obvious influence on DOC content (Figure 4b, *p* < 0.05). Briefly, the content of DOC in the rhizosphere soil without PAHs addition was 56.52 mg/kg, which increased to 59.83, 60.04, and 62.59 mg/kg, respectively, with the addition of NAP, PHE, and ANT, suggesting a DOC-mediated pathway for ROS generation. Similarly, the elevated levels of water-soluble phenols under PAH treatment, increasing from 12.55 mg/kg in the control to a range of 13.62 to 14.59 mg/kg in PAH-contaminated soils (Figure 4c), further support a phenol-mediated ROS generation pathway. In terms of enzyme activity, urease and dehydrogenase activities in PHE and ANT contaminated soils increased obviously compared with control (Figure 4d,e, *p* < 0.05), except for NAP-contaminated soil. The activity of catalase mirrored that of urease and dehydrogenase, peaking at 5.64 mg H_2_O_2_/g/20 min for soil in the presence of ANT (Figure 4f).

## 4. Discussion

### 4.1. Compound-Dependent Induction of ROS by PAHs in Rhizosphere

Three PAHs elevated rhizosphere’s ROS’ contents with distinct compound-specific effects, following the order of ANT > PHE ~ NAP (Figure 1). This differences in the enhancement strongly suggested that the molecular structure of PAHs is a critical determinant of their ability to induce rhizosphere ROS’ production. In particular, ANT, with its more conjugated ring system and higher persistence, likely intensifies plant–microbe interactions and/or alters redox-active exudate profiles, thereby driving stronger abiotic and biotic ROS production [29]. On the one hand, the high persistence and hydrophobicity of ANT may facilitate its adsorption onto plant root surfaces, which disrupts the integrity and permeability of plant root cell membranes [30,31]. This membrane damage triggers the plant’s stress response mechanism, prompting the roots to secrete more redox-active exudates, such as phenolic compounds, organic acids, and polysaccharides [32,33]. These exudates act as electron shuttles to accelerate the redox reactions in the rhizosphere, promoting the generation of ROS [34]. On the other hand, the high toxicity of ANT may reshape the rhizosphere microbial community structure; it inhibits the activity of some beneficial microorganisms while enriching functional microbes with ROS-producing capabilities. For example, *Pseudomonas* and *Bacillus* are well-documented ROS-producing, PAH-degrading microbes in the rhizoremediation literature [35,36,37], and they are hypothesized to contribute to rhizosphere ROS’ accumulation under PAH stress. Compared to previous studies that focused on single ROS species or single PAH compounds [9,12], our study is the first to quantitatively link PAHs’ molecular structure to ROS-inducing capacity, providing a predictive framework for assessing the remediation potential of different PAHs.

### 4.2. Plant Physiological Responses and Root Exudate Regulation Under PAH Stress

The enhancement of maize biomass by PAHs may induce the generation of ROS in the maize rhizosphere. The observed aboveground-promoting effect may be attributed to the plant’s compensatory growth response under contaminant stress, which often involves shifts in photosynthetic activity and resource allocation [38,39,40]. The enhancement of photosynthetic rates (Figure 2a) may be related to PAH stress, which could regulate the expression of photosynthesis-related genes in maize. Xu et al. found that PAH exposure induces increased expression of photosynthetic proteins in maize leaves [41]. Cevher-Keskin et al. also revealed the expression of photosynthesis-related genes in maize leaves; the phosphoenolpyruvate carboxylase of the C4 pathway is significantly upregulated under PAH stress [42]. Moreover, the enhanced photosynthetic efficiency under ANT stress aligns with its stronger promotion of stem elongation and ROS’ generation (Figure 1 and Figure 2b), suggesting an integrated physiological adjustment that supports biomass accumulation under PAH pressure. In contrast, the augment of net photosynthetic rate under PHE and NAP treatments was less than that in the ANT treatment, consistent with their weaker capacity to induce rhizosphere ROS’ generation.

The compositional shifts in root exudates indicated that PAHs not only enhance the total quantity of root exudation but also qualitatively restructure metabolite profiles (Figure 2b), likely reflecting differences in plant stress responses triggered by different pollutants. On the one hand, the integrated adjustments in photosynthetic activity and root exudate metabolism demonstrate how maize adapts to PAH stress. Enhanced photosynthesis supplies the carbon needed for increased exudation, while restructured exudates modulate the rhizosphere microenvironment to alleviate pollutant toxicity [43]. On the other hand, the preferential enrichment of organic acids and phenolics in root exudates had profound implications for rhizosphere ROS’ dynamics. Organic acids, with their strong chelating capacity, can mobilize soil-bound metal ions (e.g., Fe^3+^/Fe^2+^) and accelerate redox cycles, which in turn promote the generation of ROS [44,45]. This creates a positive feedback loop between exudate composition and ROS’ production. Phenolics, as redox-active molecules, can act as electron donors to mediate ROS’ production [34]. Therefore, the physiological adjustment and the exudate alterations could modulate rhizosphere processes, which may influence rhizosphere chemistry and ROS production.

### 4.3. Role of Redox Components and Enzyme Activities in ROS’ Generation

Fe(II), DOC, and water-soluble phenols, as well as the activities of catalase, urease, and dehydrogenase, are closely associated with ROS’ production [46,47]. Although bulk Fe(II) concentrations remained unchanged, this observation alone does not rule out its potential involvement in ROS’ generation. Given the complexity of Fenton chemistry, Fe(II) may still participate in ROS’ production through microscale processes (e.g., local hotspots, surface-bound species, or rapid redox cycling) that are not captured by bulk measurements. Future studies employing spatially resolved techniques (e.g., nanoparticle-based sensors or planar optodes) are needed to clarify the role of Fe(II) in rhizosphere ROS’ production [47]. In contrast, the significant increases in DOC content suggest a DOC-mediated pathway for ROS’ generation. DOC serves as both a source of electron donors for abiotic ROS formation and a substrate for microbial metabolism that can lead to biotic ROS’ production [48,49]. The alignment of DOC enhancement order (ANT > PHE ~ NAP) with the compound-specific ROS pattern suggests that DOC is a key contributor to the differences in ROS-inducing capacities among PAHs. Similarly, the elevated levels of water-soluble phenols further support a phenol-mediated ROS generation pathway, as phenolic compounds are known to participate in redox cycling, acting as electron shuttles that reduce molecular oxygen to generate O_2_^•−^ and subsequently H_2_O_2_ and ^•^OH [9,50].

The significant increases in urease and dehydrogenase activities under PHE and ANT treatments indicate enhanced microbial metabolic activity in response to PAH stress. This stimulation of microbial activity aligns with the observed increases in DOC and water-soluble phenols, suggesting that PAH stress promotes the release of labile carbon substrates that serve as energy sources for microbial communities [51]. Notably, the increased activity of dehydrogenase, an intracellular enzyme involved in respiratory electron transport chains, suggested enhanced microbial electron transfer activity under PAH stress [52]. This elevated electron flow could drive biotic ROS’ production through microbial metabolic processes, including the reduction of molecular oxygen via respiratory chains or the activity of oxidoreductases [35]. However, it is important to emphasize that these inferences are based on enzyme activity measurements, which serve as proxies for microbial activity, rather than for direct characterization of microbial community composition, abundance, or functional gene expression. The parallel increase in catalase activity indicated an adaptive microbial response to elevated oxidative stress. Catalase serves as a key antioxidant enzyme that scavenges H_2_O_2_, thereby protecting microbial cells from oxidative damage [53]. The enhanced catalase activity under PAH stress suggests that microorganisms are actively responding to elevated levels of ROS by upregulating their antioxidant defense systems.

### 4.4. Possible Mechanisms of Rhizosphere ROS’ Production Under PAH Stress

**Figure 5:**
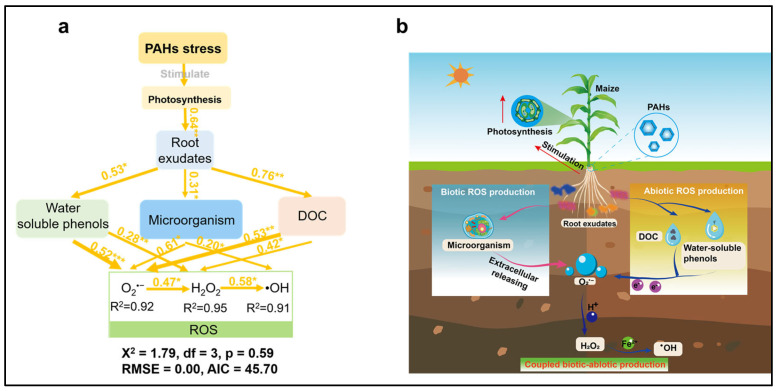
(**a**) Structural equation modeling of production of rhizosphere reactive oxygen species (ROS) under stress of PAHs (χ^2^ = 1.79, degrees of freedom = 3, probability level = 0.59, and RMSE = 0.00). Orange solid arrows indicate positive correlation, and arrow thickness is proportional to association intensity. *, *p* < 0.05; **, *p* < 0.01; ***, *p* < 0.001. (**b**) Schematic diagram of potential mechanism of rhizosphere ROS’ generation.

To explore the potential mechanism of ROS’ generation under PAH stress, we first employed correlation analysis to probe critical factors influencing ROS’ generation. As presented in Figure S2, significant correlations were observed between ROS’ levels and root exudates, DOC, water-soluble phenols, and enzyme activities, further indicating that PAHs stress may mediate the generation of rhizosphere ROS by modulating root exudate composition and quantity, water-soluble phenol content, DOC concentration, and microbial activity [54]. To further explore the pathways underlying ROS’ generation under PAH stress in the rhizosphere, SEM analysis was conducted. As exhibited in Figure 5a, SEM explained 92.0%, 95.0% and 91.0% of the variability in O_2_^•−^, H_2_O_2_, and ^•^OH generation under PAH stress, providing a superb fit using the χ^2^, RMSE and AIC metrics. The obtained results suggest that PAHs stress could first stimulate photosynthesis in maize, which regulates the quantity and composition root exudates and then influences the production of water-soluble phenols and DOC and the reproduction of microorganisms, thereby indirectly facilitating the abiotic and biotic generation of ROS.

Overall, the production of rhizosphere ROS in PAH-contaminated soil may be attributed to the following processes (Figure 5b). PAH exposure promotes the growth and photosynthesis of maize, which in turn increase the release of root exudates. Root exudates influence O_2_^•−^ generation in two aspects. On the one hand, root exudates could provide carbon and energy sources for microorganisms, driving the biotic production of O_2_^•−^ via extracellular secretion [35,37,55]. On the other hand, as the primary redox-metastable components of root exudates, water-soluble phenols and DOC could facilitate electron donation to oxygen, contributing to abiotic O_2_^•−^ generation [9]. The generated O_2_^•−^ readily undergoes dismutation and hydrolysis to yield H_2_O_2_ [56], which may be subsequently decomposed via Fe(II)-mediated Fenton reactions to generate ^•^OH [57]. These findings revealed that the coupled biotic–abiotic processes elicited by PAH stress acted a critical role in driving rhizosphere ROS’ generation. However, it should be noted that this proposed pathway is currently inferred from the literature rather than from direct kinetic measurements. To further validate the proposed Fenton pathway, future studies should perform kinetic experiments to quantify Fe(II) consumption and ^•^OH generation rates. Moreover, it is important to note that the mechanistic pathway proposed herein—from PAH stress to enhanced photosynthesis, root exudation, and ROS’ generation—is inferred primarily from correlational analyses and temporal concordance. While these observations are consistent with the hypothesized sequence, our experimental design does not allow for definitive causal attribution. Future studies employing targeted interventions (e.g., photosynthetic inhibitors, root-exudate-collection mutants, or isotope-labeling approaches) are warranted to rigorously test this causal framework.

### 4.5. Implications

To date, the generation and regulatory mechanisms of rhizosphere ROS under pollutant exposure remain poorly understood. This study systematically characterized, for the first time, the generation patterns of O_2_^•−^, H_2_O_2_, and ^•^OH in the maize rhizosphere under NAP, ANT and PHE stress, together with the corresponding variations in physicochemical and biochemical rhizosphere properties. Our results revealed that rhizosphere ROS levels displayed a distinct compound-specific pattern under PAH exposure, following the order ANT > PHE ~ NAP. This pattern was closely coupled with rhizosphere processes including root exudates, DOC content, water-soluble phenol production, and soil enzyme activities. These results establish a novel theoretical foundation for the scientific evaluation and risk assessment of soils contaminated with different PAHs. Moreover, this study clarified the regulatory pathway of ROS’ generation in the rhizosphere under PAH stress. Specifically, PAHs enhance photosynthetic efficiency by promoting maize growth, drive an increase in the release of root exudates, and further synergistically strengthen the abiotic and biotic generation processes of ROS through stimulating the release of water-soluble phenols and DOC, as well as promoting the proliferation of rhizosphere microorganisms. This result provides support for soil remediation technologies based on rhizosphere ROS’ regulation. By adjusting crop photosynthetic efficiency, optimizing the composition of root exudates, or directionally enriching functional microbial communities, the in situ rhizosphere ROS’ production capacity can be strengthened [9]. This, in turn, boosts the degradation efficiency of persistent organic pollutants like PAHs, thereby laying a scientific groundwork for the development of low-cost, eco-friendly phytoremediation technologies.

## 5. Conclusions

Our findings suggested that three PAHs significantly increased the O_2_^•−^, H_2_O_2_, and ^•^OH contents in the maize rhizosphere, with a compound-specific promotion effect following the order of ANT > PHE ~ NAP. PAHs may enhance photosynthetic efficiency by promoting maize growth, facilitate the secretion of root exudates, and further synergistically strengthen the abiotic and biotic generation processes of ROS through stimulating the release of water-soluble phenols and DOC, as well as promoting microbial activity. These findings improve our understanding of rhizosphere ROS’ responses to PAH stress, as well as their associations with plant physiological traits and the rhizosphere’ environmental factors. Moreover, our results provide clear guidance to enhance the in situ phytoremediation of PAH-contaminated soils. Maize could be prioritized for remediating PAH-polluted soils, especially sites contaminated with high-molecular-weight PAHs like anthracene, given its strong capacity to boost rhizosphere ROS’ production. Meanwhile, agronomic measures that stimulate maize photosynthesis and the secretion of organic acids and phenolic root exudates (e.g., water and nutrient management) should be adopted to amplify rhizosphere biotic–abiotic ROS’ generation and accelerate PAH degradation.

Nevertheless, several limitations should be acknowledged. This work relies on a single PAH concentration and a single 24-day sampling time point under controlled pot conditions, which cannot fully replicate complex field soil matrices with mixed PAH pollutants, fluctuating moisture or competing native vegetation. We only quantified total rhizosphere ROS without partitioning the relative contributions of biotic versus abiotic ROS’ generation, and full taxonomic profiling of rhizosphere microbial communities was not performed to pinpoint ROS-generating functional taxa. Future work should establish PAH dose–response curves to identify threshold concentrations that alter rhizosphere ROS’ dynamics. Field-scale validation across different soil textures and pollution levels is needed to translate pot-based findings into agricultural practice. High-throughput sequencing will be used to characterize shifts in rhizosphere microbial functional groups associated with ROS’ metabolism. Additionally, isotopic tracing and quenching assays will quantify the direct contribution of rhizosphere ROS to PAH degradation, to build quantitative frameworks linking rhizosphere redox activity to remediation efficiency.

## Data Availability

Data are contained within the article and Supplementary Materials.

## Supplementary Materials

The following supporting information can be downloaded at: https://www.mdpi.com/article/10.3390/life16071136/s1, Text S1–Text S8: Details about the experimental reagents, and analytical protocols; Table S1. Changes in root exudates types in maize under PAHs stress; Table S2: Changes in amount of root exudates in maize under PAHs stress; Figure S1: Fluorescence imaging displayed the hotspot of ROS generation in the root-soil interface; Figure S2: The correlation analysis among ROS, photosynthetic activity, root exudates, Fe(II), DOC, water-soluble phenols, and enzymes activities. References [27,28,58] are cited in the Supplementary Materials.
