# Supplementary material for: Unraveling Polycyclic Aromatic Hydrocarbon-Triggered Reactive Oxygen Species’ Generation in Maize Rhizosphere: Coupled Biotic–Abiotic Mechanism
Source: Life (Basel). 2026 Jul 8;16(7):1136. doi: 10.3390/life16071136 (PMC13413345; doi:10.3390/life16071136)
Supplement: Supplementary file 1 [file life-16-01136-s001.zip › life-4394421-supplementary.pdf]

Supplementary material

Unraveling Polycyclic Aromatic Hydrocarbon-Triggered Reactive Oxygen Species Generation in the Maize Rhizosphere: A Biotic–Abiotic Coupling Mechanism

Xiaoling Xu[a], Chuanxiang Li[a], Jinbo Liu[a]*, Jian He[a], Yongxiu Sun[a,b], Jian Wang[a]

[a] School of Petroleum Engineering and Environmental Engineering, Yan’an University, Yan’an 716000, China

[b] Yan’an key laboratory of Ecological Restoration and Carbon Sequestration Regulation, Yan'an, 716000, China

* Corresponding author E-mails: liujinbo@yau.edu.cn

Text S1 Naphthalene (NAP, 98%), anthracene (ANT, 98%), phenanthrene (PHE, 98%), benzoic acid (BA, 99%), p-hydroxybenzoic acid (*p*-HBA, 98%), vanillic acid (98%) and 1,10-o-phenanthroline (98%) were all supplied by J&K Chemical Ltd. (Beijing, China). 2′,7′-dichlorohydro fluorescein diacetate (DCFH-DA, > 97%) was obtained from Aladdin Biochemical Technology Co., Ltd., Shanghai, China. 2,3-bis(2-methoxy-4-nitro-5-sulfophenyl)-2H-tetrazolium-5-carboxanilide (XTT, 98%) was purchased from Sigma Co. Ltd. (Shanghai, China). Methanol (HPLC grade) and phosphoric acid (H_3PO_4, > 85%, AR) were obtained from Sinopharm Chemical Reagent Co., Ltd. (Shanghai, China). All chemicals were used without further purification.

Text S2 The root exudates of maize were measured according to the previous method proposed by Jiang et al. (2021) with some modification [1]. Specifically, the maize was shaken carefully until the soils detached from roots. After that, the obtained maize was cultured in sterile water for 12 h. The water containing root exudates was freeze-dried to ∼10 mL and filtered using a 0.22 μm nylon membrane. Then, the extracts were dried under N_2 gas and then methoximated and trimethylsilylated. The exudates were analyzed by an GC−MS (GCMS-TQ8050 NX, Japan). The capillary column used was a DB-5MS (30 m × 0.25 mm i.d. × 0.25 μm film thickness). The GC injection temperature was 50 °C ramped to 250 °C by 12 °C s^{-1}, and the column temperature was first set at 50 °C for 1 min, then ramped at 20 °C min^{-1} to 330 °C and finally held constant for 5 min. The mass spectrometer was used with unit mass resolution at 17 spectra s^{-1} from 80 to 500 Da at −70 eV ionization energy and 1800 V detector voltage with a 230 °C transfer line and a 250 °C ion source.

Text S3 XTT was used to quantify $O_2^{\bullet-}$ based on its reaction with $O_2^{\bullet-}$ to produce XTT formazan (Fu et al., 2016) [2]. Briefly, 0.05 g soil samples were mixed with 5 mL 0.05 mM XTT solution and shaken for 24 h under dark condition. Subsequently, the mixture was centrifuged for 5 min at 7000 ×*g*, and the obtained supernatants were collected and filtered using a syringe equipped with a 0.45 μm nylon organic membrane. Finally, the XTT formazan in the filtrate was determined by an Evolution 201 UV-Visible spectrophotometer (Thermo Scientific, USA) at 475 nm ($\varepsilon = 23{,}800\ M^{-1}\ cm^{-1}$).

H_2O_2 concentration was determined by hydrogen peroxide assay kit purchased from Solarbio Life Sciences Co., Ltd (Beijing, China). Briefly, 0.05 g soil samples were mixed with ultrapure water to achieve a suspension of 10 g/L. After that, the suspension was vortexed for 5 min and then centrifuged for 5 min at 7000 ×*g*. The obtained supernatants were collected and filtered using a syringe equipped with a 0.45 μm nylon water membrane. Finally, the filtrate was utilized for H_2O_2 analysis following the manufacturers' protocol.

Benzoic acid (BA), applied as a chemical probe to quantitatively detect •OH concentration, can react with •OH ($k_{BA,\bullet OH} = 5.9 \times 10^9\ M^{-1}S^{-1}$) and transformed to *p*-HBA [3]. Specifically, 0.05 g soil samples were mixed with 5 mL 10 mM BA solution and shaken for 24 h under dark condition. Subsequently, the mixture was centrifuged for 5 min at 7000 ×g, and the obtained supernatants were collected and filtered using a syringe equipped with a 0.45 μm nylon organic membrane. Finally, the filtrate was placed in amber vial and analyzed for the concentration of p-HBA using a high performance liquid chromatography (HPLC-UV, Thermo Fisher Scientific Ulttimate-3000, USA) equipped with an ultraviolet (UV) detector and an Inter Sustain C18

column (4.6 × 250 mm^2). A sample volume of 20 μL was injected into the HPLC by an autosampler. HPLC conditions were: 85% methanol and 15 % ultrapure water as mobile phase; flow rate of 1 mL min^{-1}; oven temperature 30 °C; UV wavelength of *p*-HBA at 255 nm; total run time was 10 min.

Text S4 Fe(II) in the rhizosphere soil was determined by 1,10-o-phenanthroline analytical method. Specifically, 0.05 g soil was immersed in 5 mL of 0.1 M HCL and rotated at 200 rpm for 2 h. After that, the mixture was centrifuged at 7000 × g for 10 min, and the supernatant was filtered through a 0.45μm filter membrane. Finally, the concentration of Fe(II) was determined by a 1,10-*o*-phenanthroline analytical method at 510 nm using an ultraviolet-visible (UV–vis) spectrophotometer (Evolution 201, Thermo).

Text S5 The rhizosphere soil was mixed with deionized water at a ratio of 1: 10000 (w/v) and shaken for 24 h. After that, the mixture was centrifuged at 5000 × g for 10 min, and the supernatant was filtered through a 0.45μm filter membrane. Finally, the extract was determined by total organic carbon analyzer (TOC-L, Shimadzu, Japan).

Text S6 Water-soluble phenols content was quantified colorimetrically using Folin–Ciocalteau reagent. Specifically, 0.05 g rhizosphere soils were extracted by shaking with 25 mL distilled water for 4 h at 100 rpm, followed by centrifugation for 15 min at 5000 rpm. A 10-mL aliquot of extract or standard was placed in a test tube, and 3 mL of Na_2CO_3 solution and 1 mL of Folin-Ciocalteau reagent were added. The solution was mixed well and stand for 1 h at room

temperature, and the absorbance of the blue complex formed during this period was determined spectrometrically at 750 nm. Vanillic acid was used as standard, and the amount of phenolic compounds was expressed as vanillin acid equivalents (μg vanillic acid/g soil).

Text S7

Urease activity: urease activity was measured by the sodium phenate-sodium hypochlorite colorimetric method using urea as the substrate. 0.05 g soil samples were placed in a 50 mL Erlenmeyer flask containing 1 mL of toluene, and the mixture was shaken on a mechanical shaker for 15 min. After that, 10% urea solution and 20 mL citrate buffer solution (pH = 6.7) were added to the mixture. The mixture was shaken well and incubated in a 37 °C incubator for 24 h. After incubation, the soil suspension was filtered using quantitative filter paper, and the obtained filtrate (1 mL) were transferred into 50 mL volumetric flask containing 4 mL of 1.35 M sodium phenol solution and 3 mL of 0.9% sodium hypochlorite solution. After reaction for 20 min, ultrapure water was added to the mark of the volumetric flask. Finally, the absorbance of the blue complex formed during this period was determined spectrometrically at 578 nm. Nitrogen standard solution was used as standard, and soil urease activity was expressed as mg NH_4-N/g/24 h.

Dehydrogenase activity: Dehydrogenase activity was analyzed by measuring the reduction of triphenyl-tetrazolium chloride to triphenyl-formazan. 0.05 g soil samples were incubated for 24 h at 37 ºC in 5 mL of 2,3,5-triphenyltetrazolium chloride (TTC) solution. Two drops of concentrated H_2SO_4 were added immediately after the incubation to each sample in order to stop the reaction. The sample was then blended with 5 mL of toluene to extract the reaction

product (i.e., TPF) and shaken for 30 min at 250 rpm, followed by centrifugation at 5000 rpm for 5 min and spectrophotometric analysis of absorbance at 492 nm. Soil dehydrogenase activity was expressed as mg TPF /g dry soil/24 h.

Catalase activity: Catalase was assayed as described by Aebi, whereby the activity was determined based on the degradation of H_2O_2. 0.05 g soil samples were placed in a 100 mL Erlenmeyer flask containing 40 mL distilled water and 5 mL of 0.3% H_2O_2. The mixture was shaken on a mechanical shaker for 20 min. After that, 1 mL saturated aluminum potassium alum solution was added to the mixture immediately. The soil suspension was filtered to a 50 mL Erlenmeyer flask containing 5 mL of 1.5 M H_2SO_4. After filtering, the absorbance of the filter was determined spectrometrically at 240 nm.

Text S8

Sample size for SEM analysis: The SEM dataset was built upon 36 independent biological samples (4 treatments × 9 replicate pots). Each data point corresponds to averaged physiological, root exudate and rhizosphere ROS measurements from one individual pot, meeting the conventional sample-size threshold for stable SEM fitting.

Model identification procedures: We followed a two-step modeling strategy. (1) Measurement model construction: All observed variables were grouped into latent factors (plant photosynthetic traits, root exudate fractions, rhizosphere redox indicators) based on prior rhizosphere mechanistic literature. (2) Structural model modification: Initial saturated model was simplified by removing non-significant pathways one by one to achieve an over-identified framework. No correlated error terms were arbitrarily added unless justified by biological

relevance. We report the degrees of freedom (df) of the final optimized model to confirm valid identification.

Standardized path coefficients: All standardized path coefficients (β values) with corresponding p-values are explicitly presented in the revised SEM figure. Solid lines mark significant pathways ($p < 0.05$), while dashed lines represent non-significant links that were excluded from the final optimal model.

Table S1. Changes in root exudates types in maize under PAHs stress.

Samples	Ester	Sugar	Alcohol	Organic acids	Amino acids	Phenol	Others	Type of compound
CK	7	3	3	4	2	4	42	65
NAP	8	1	3	4	2	6	51	75
PHE	7	2	4	6	2	5	57	83
ANT	4	2	3	8	2	7	64	90

Table S2. Changes in amount of root exudates in maize under PAHs stress.

Samples	Ester	Sugar	Alcohol	Organic acids	Amino acids	Phenol	Others	Compound quantity
CK	12	8	6	11	3	4	40	84
NAP	14	5	4	15	2	9	67	116
PHE	15	6	7	18	3	10	75	134
ANT	15	10	7	24	2	9	84	151

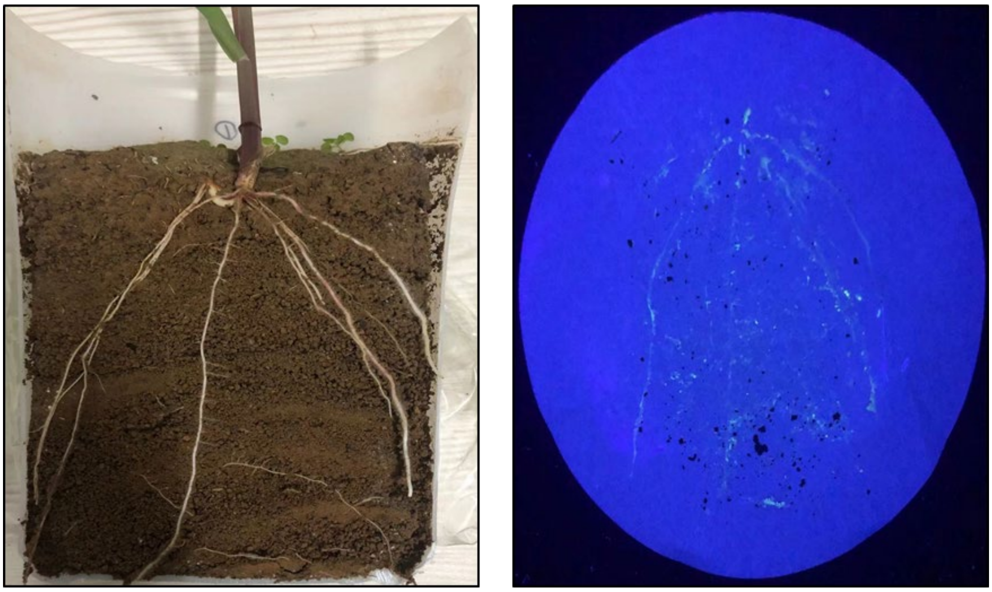

Figure S1. Fluorescence imaging displayed the hotspot of ROS generation in the root-soil interface

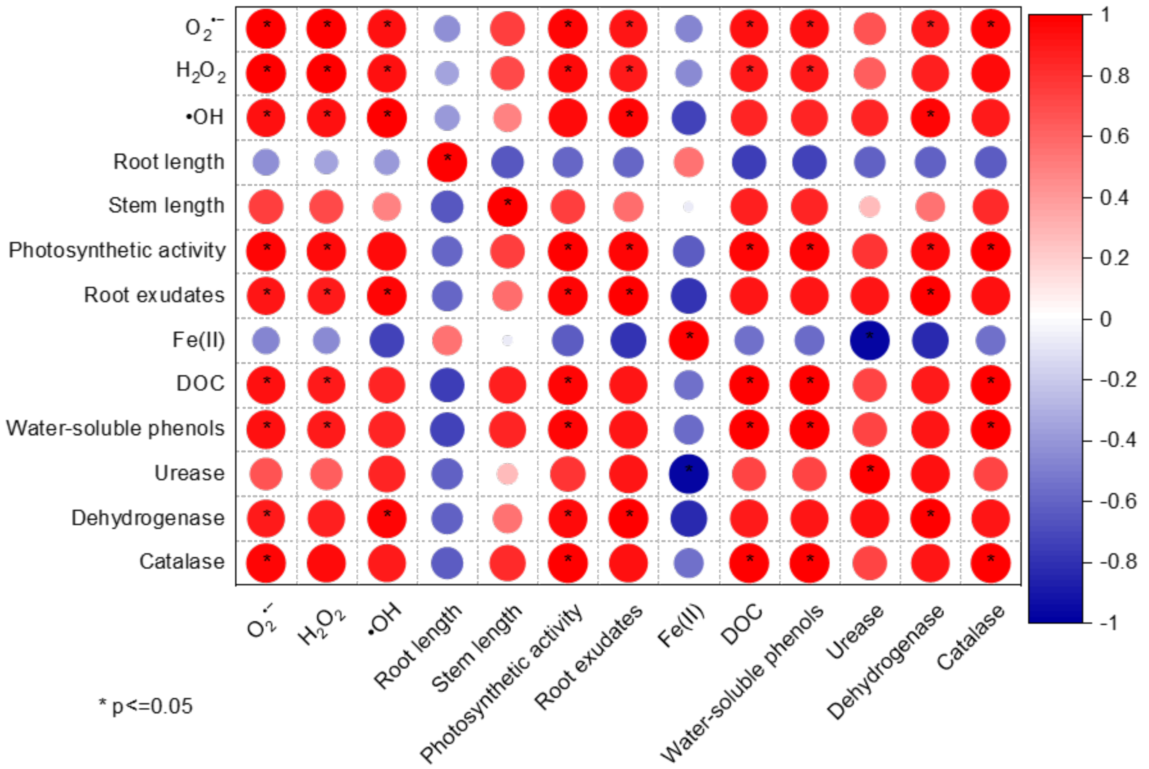

Figure S2. The correlation analysis among ROS, photosynthetic activity, root exudates, Fe(II), DOC, water-soluble phenols, and enzymes activities.

References

1. Jiang, L.; Luo, C.; Zhang, D.; Song, M.; Mei, W.; Sun, Y.; Zhang, G. Shifts in a phenanthrene-degrading microbial community are driven by carbohydrate metabolism selection in a ryegrass rhizosphere. *Environ. Sci. Technol.* **2021**, *55*, 962−973.

2. Fu, H.; Liu, H.; Mao, J.; Chu, W.; Li, Q.; Alvarez, P.J.J.; Qu, X.; Zhu, D. Photochemistry of dissolved black carbon released from biochar: reactive oxygen species generation and phototransformation. *Environ. Sci. Technol.* **2016,** *50*, 1218–1226.

3. Zhang, P.; Yuan, S.; Liao, P. Mechanisms of hydroxyl radical production from abiotic oxidation of pyrite under acidic conditions. *Geochim. Cosmochim. Acta* **2016**, *172*, 444–457.
